# DMV extrasynaptic NMDA receptors regulate caloric intake in rats

**DOI:** 10.1172/jci.insight.139785

**Published:** 2021-05-10

**Authors:** Courtney Clyburn, R. Alberto Travagli, Amy C. Arnold, Kirsteen N. Browning

**Affiliations:** Department of Neural and Behavioral Sciences, Penn State College of Medicine, Hershey, Pennsylvania, USA.

**Keywords:** Gastroenterology, Neuroscience, Ion channels, Obesity, Synapses

## Abstract

Acute high-fat diet (aHFD) exposure induces a brief period of hyperphagia before caloric balance is restored. Previous studies have demonstrated that this period of regulation is associated with activation of synaptic N-methyl-D-aspartate (NMDA) receptors on dorsal motor nucleus of the vagus (DMV) neurons, which increases vagal control of gastric functions. Our aim was to test the hypothesis that activation of DMV synaptic NMDA receptors occurs subsequent to activation of extrasynaptic NMDA receptors. Sprague-Dawley rats were fed a control or high-fat diet for 3–5 days prior to experimentation. Whole-cell patch-clamp recordings from gastric-projecting DMV neurons; in vivo recordings of gastric motility, tone, compliance, and emptying; and food intake studies were used to assess the effects of NMDA receptor antagonism on caloric regulation. After aHFD exposure, inhibition of extrasynaptic NMDA receptors prevented the synaptic NMDA receptor–mediated increase in glutamatergic transmission to DMV neurons, as well as the increase in gastric tone and motility, while chronic extrasynaptic NMDA receptor inhibition attenuated the regulation of caloric intake. After aHFD exposure, the regulation of food intake involved synaptic NMDA receptor–mediated currents, which occurred in response to extrasynaptic NMDA receptor activation. Understanding these events may provide a mechanistic basis for hyperphagia and may identify novel therapeutic targets for the treatment of obesity.

## Graphical abstract

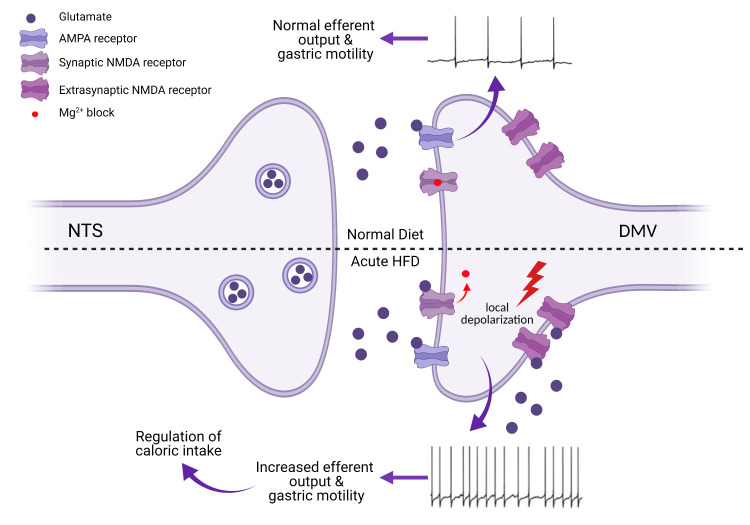

## Introduction

The rates of obesity and its comorbid disorders, including hypertension, type 2 diabetes, and heart disease, have been increasing dramatically in the United States and worldwide (1), highlighting the importance of understanding the neural mechanisms involved in the regulation of visceral functions, such as feeding and digestion. Obesity is a complex multifactorial disorder composed of genetic, epigenetic, environmental, and behavioral factors, but ultimately occurs when energy intake exceeds expenditure (2–5). The gastrointestinal (GI) tract is one of several organs that contribute to the peripheral signaling of food intake and satiety. Increasingly, plasticity within vagally mediated GI functions is recognized as playing an important role in the neural regulation of energy balance. Sensory inputs from the stomach and upper GI tract transmit chemical and mechanical information through vagal afferent fibers to the neurons of the nucleus tractus solitarius (NTS; refs. 6, 7). The NTS integrates this sensory signal with inputs from the brainstem and hypothalamus involved in energy regulation and sends either glutamatergic, GABAergic, or catecholaminergic projections to the adjacent dorsal motor nucleus of the vagus (DMV). The DMV provides parasympathetic efferent (motor) output to the stomach and upper GI tract to coordinate gastric functions, food intake, and energy homeostasis. (6, 7).

Several studies have shown that prolonged high-fat diet (HFD) exposure and diet-induced obesity have profound effects on vagal sensory functions, reducing afferent excitability and responsiveness (8–14). Less attention has been paid, however, to the neuroplasticity that occurs in response to acute HFD (aHFD) exposure. Studies in humans and rodents have demonstrated that in the acute period after HFD exposure, a brief (24 hour) period of hyperphagia occurs before control over caloric intake is restored within 3 to 5 days (14–17). Previous studies have also determined that hindbrain glutamatergic N-methyl-D-aspartate receptors (NMDARs) play a critical role in meal termination, as well as the cholecystokinin-induced (CCK-induced) decrease in food intake (18–20). Notably, we have shown this period of caloric regulation is associated with an increase in activation of synaptic NMDARs on DMV neurons, increasing neuronal excitability, and vagal efferent output regulating gastric tone and motility (14). It remains to be determined, however, whether the activation of DMV synaptic NMDARs is required for or simply associated with the homeostatic regulation of caloric intake, and the mechanisms responsible for this upregulation in NMDAR-mediated glutamatergic signaling have still to be elucidated.

NMDARs are glutamatergic ionotropic heterodimers typically composed of 2 GluN1 and either 2 GluN2 or GluN3 subunits (21, 22). Although still somewhat controversial, the subunit composition of these receptors is generally considered to determine their location: extrasynaptic NMDARs are enriched with GluN2B subunits, whereas synaptic NMDARs are enriched with GluN2A subunits (21, 22). Under basal physiological conditions, glutamatergic transmission to DMV neurons occurs via AMPA receptor activation, with little or no observable synaptic NMDAR–mediated response (14, 23), likely due to the voltage-dependent Mg^2+^ gating block of ionic currents at resting membrane potentials (14, 24). After aHFD exposure, however, glutamatergic transmission activates AMPA and synaptic NMDARs, and the temporal pattern of the upregulation of glutamatergic (synaptic NMDAR–) signaling is associated with the restoration of caloric balance. Previous studies in other brain regions have shown that activation of extrasynaptic NMDARs can cause a significant local depolarization sufficient to remove the Mg^2+^-dependent inactivation block of synaptic NMDARs (25, 26). The aim of the present study, therefore, was to assess the mechanistic basis of synaptic NMDAR activation on DMV neurons, whether this requires the activation of extrasynaptic NMDARs, and if plasticity with brainstem glutamatergic signaling is required for the regulation of caloric balance after aHFD exposure.

## Results

Each electrophysiology experiment included recordings from corpus- and antrum/pylorus-labeled neurons as well as recordings from male and female rats. As shown previously (14), there were neither qualitative nor quantitative differences in the glutamate-mediated electrophysiological responses of corpus- and antrum/pylorus-labeled DMV neurons or between male and female rats. All results were therefore combined.

### Selectivity of extrasynaptic NMDAR antagonists in DMV neurons.

Antagonists of extrasynaptic NMDARs have been shown previously to have variable selectivity for synaptic NMDARs (27–29), and memantine in particular may antagonize α7-containing nicotinic receptors (30) and 5-HT3 receptors (31), which may modulate central vagal neurocircuits (32–39). Therefore, we conducted a series of experiments in which the effects of the extrasynaptic NMDAR antagonist, memantine (30 μM), as well as the NR2B subunit–containing NMDA receptor (primarily extrasynaptic) antagonist, ifenprodil (3 μM, ref. 40), were assessed on evoked (synaptic) NMDAR-mediated currents.

In DMV neurons voltage-clamped at +20 mV, glutamatergic currents were evoked via electrical stimulation of the adjacent NTS. In the presence of the AMPAR antagonist, 6,7-dinitroquinoxaline-2,3-dione (DNQX) (30 μM), the remaining NMDAR-dependent current (14) was unaffected by either memantine or ifenprodil. Specifically, the amplitude, decay time, and area of evoked excitatory postsynaptic currents (eEPSCs) were unaffected by memantine in 5/5 control DMV neurons from 3 rats (108.6% ± 4.51%, 93.1% ± 2.49%, and 105.3% ± 5.22%, respectively; *P >* 0.05 for each) and 6/7 aHFD DMV neurons from 3 rats (101.8% ± 4.51%, 101.9% ± 4.83%, and 95.6% ± 5.98%, respectively; *P >* 0.05 for each; Supplemental Table 1 and Supplemental Figure 1; supplemental material available online with this article; https://doi.org/10.1172/jci.insight.139785DS1). Similarly, ifenprodil had no effect on eEPSC amplitude, decay time, or area in 5/5 control DMV neurons from 3 rats (100.9% ± 8.29%, 91.4% ± 1.97%, and 105.9% ± 11.76%, respectively; *P >* 0.05 for each) or in 5/5 aHFD DMV neurons from 3 rats (108.7% ± 6.91%, 99.4% ± 4.03%, and 85.0% ± 2.94%, respectively; *P >* 0.05 for each; Supplemental Table 1 and Supplemental Figure 1). These results confirmed that at the concentrations used, memantine and ifenprodil had no effect on synaptic currents or synaptic NMDARs.

### aHFD-induced activation of synaptic NMDARs is dependent upon activation of extrasynaptic NMDARs.

Previously, we demonstrated that synaptic NMDAR–mediated currents were not present after 1 day of aHFD exposure when rats were hyperphagic, but that synaptic NMDAR–mediated synaptic transmission was uncovered when caloric balance was restored after 3 to 5 days of aHFD (14). To assess whether this diet-induced activation of synaptic NMDARs is dependent upon extrasynaptic NMDAR activation, whole-cell patch-clamp recordings were made from gastric-projecting DMV neurons in thin brainstem slices of control and aHFD rats.

### Synaptic NMDARs are not activated in control DMV neurons.

**Figure 1:**
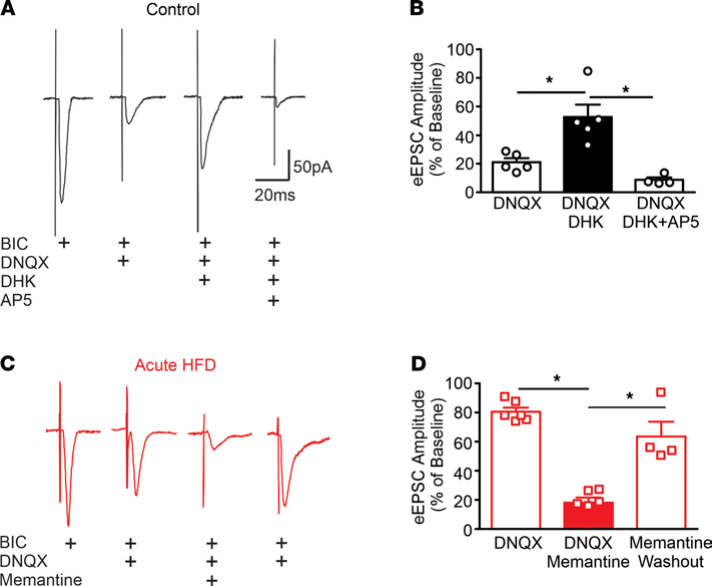
Activation of extrasynaptic NMDARs is required for the activation of synaptic NMDARs observed after aHFD exposure.(A and C) Representative traces (averaged from 6–10 raw traces) of eEPSCs from control (A) and aHFD (C) gastric-projecting DMV neurons voltage-clamped at –50mV. In control conditions (**A**), application of DNQX significantly reduced eEPSC amplitude, which was recovered after application of DHK. This NMDA-mediated current was then reduced by application of AP5. After aHFD (**C**), application of DNQX did not significantly affect eEPSC amplitude. The remaining NMDA-mediated current was significantly and reversibly decreased after application of memantine. (**B** and **D**) Graphical summary of the effects of DNQX, DHK, memantine, and AP5 on eEPSC amplitude in control (**B**) and aHFD (**D**) gastric-projecting DMV neurons (**B**; *n =* 5 cells, 3 rats) (**D**; *n =* 6 cells, 3 rats). In controls, application of DNQX (left; open bars) significantly reduced eEPSC amplitude. Subsequent application of DHK increased eEPSC amplitude (middle; black bar), and this synaptic NMDA-mediated current was reduced after subsequent application of AP5 (right; open bars). After aHFD (**D**), application of DNQX (left; open bar) did not alter eEPSC amplitude. Subsequent application of memantine significantly reduced eEPSC amplitude (middle; red bar), which was reversed after washout (right; open bar). *P < 0.05 (1-way ANOVA with post hoc Bonferroni test).

In control DMV neurons voltage-clamped at –50mV, application of the AMPAR antagonist, DNQX (30 μM) reduced the amplitude of eEPSCs significantly (baseline 149 ± 15.4 pA versus DNQX 30 ± 3.0 pA; *P <* 0.05; *n =* 5 neurons, 3 rats; Figure 1, A and B), confirming the majority of the glutamatergic current in control conditions was AMPA-mediated (14). To investigate whether exogenously stimulating extrasynaptic NMDARs could uncover synaptic NMDAR–mediated currents, the glutamate uptake inhibitor dihydrokinate (DHK; 30 μM) was used to block astrocytic glutamate uptake. In the continued presence of DNQX, application of DHK partially recovered the amplitude of eEPSCs (77 ± 13.5 pA; *P <* 0.05 versus DNQX alone), an increase that was sensitive to application of the synaptic NMDAR–selective antagonist, (2R)-amino-5-phosphonopentanoate (AP5) (25 μM; 14 ± 4.4 pA; *P <* 0.05 versus DNQX plus DHK; Figure 1, A and B), suggesting that activation of extrasynaptic NMDARs did, indeed, uncover a significant synaptic NMDAR–mediated current under control conditions.

**Figure 2:**
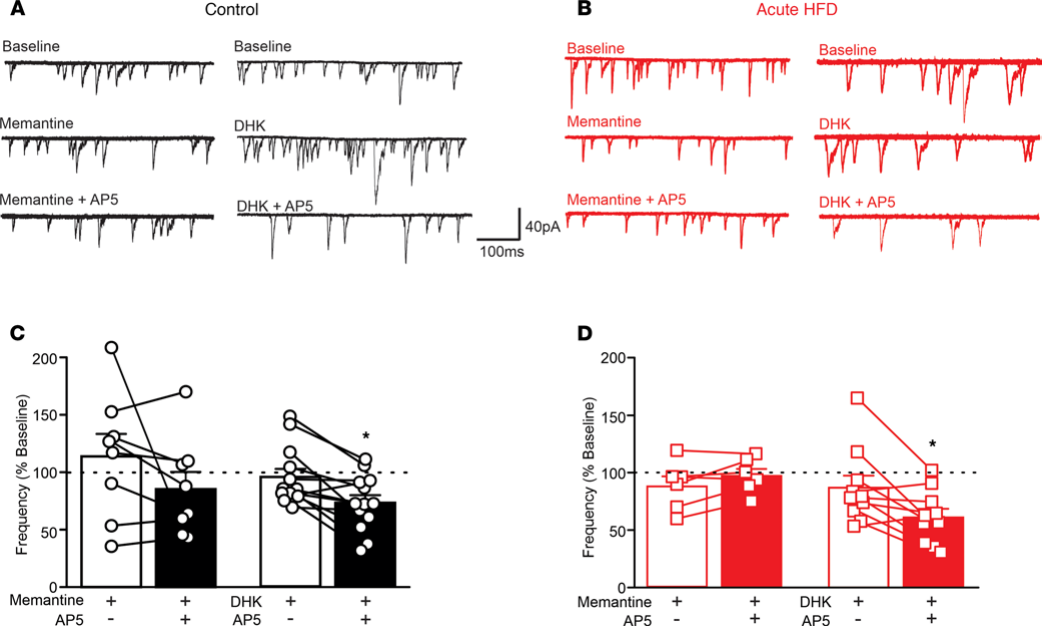
Inhibition of extrasynaptic NMDARs attenuates the synaptic-mediated decrease in glutamatergic currents observed after aHFD exposure, whereas stimulation of extrasynaptic NMDARs uncovers this effect in control conditions. (**A** and **B**) Six overlapping consecutive traces from gastric-projecting control (**A**) and aHFD (**B**) DMV neurons voltage-clamped at –50mV illustrating mEPSCs. In controls (**A**), application of memantine (30 μM; left, middle) or AP5– (25 μM; left, bottom) had no effect on mEPSC frequency. Application of DHK (30 μM; right, middle), however, uncovered an AP5-mediated decrease (25 μM; right, bottom) in mEPSC frequency. After aHFD (**B**), memantine (30 μM; middle) attenuated the AP5-mediated decrease (25 μM; bottom) in mEPSC frequency. Application of DHK had no significant effect on mEPSC frequency (30 μM; right, middle) and did not affect the AP5-mediated decrease in mEPSC frequency. (**C** and **D**) Graphical summary of the effects of memantine (**C**; left; *n =* 8 cells, 5 rats) (**D**; left; *n =* 6 cells, 3 rats) and DHK (**C**; right; *n =* 12 cells, 4 rats) (**D**; right; *n =* 10 cells, 3 rats) on the AP5-mediated changes in mEPSC frequency in control conditions (**C**) and after aHFD (**D**). In controls (**C**), memantine (left) had no significant effect on AP5-mediated changes mEPSC frequency in control conditions (left). DHK (middle), however, uncovered a significant AP5-mediated decrease in mEPSC frequency. Note that AP5 alone had no significant effect on frequency of mEPSCs in controls (*n =* 6 cells, 3 rats). After aHFD exposure (**D**), DHK (right) had no effect on the AP5-mediated decrease in mEPSC frequency. Memantine (left), however, significantly attenuated the AP5-mediated decrease normally observed after aHFD exposure. Note that the effect size of AP5 alone was similar to that of AP5 following DHK (right; *n =* 6 cells, 3 rats). Baseline represented by dashed line (100%). *P < 0.05 versus DHK or memantine alone or versus baseline (Student’s paired *t* test).

To further investigate the role that extrasynaptic NMDAR–mediated currents play in the activation of synaptic NMDAR activation, the ability of AP5 to inhibit miniature EPSCs (mESPCs; i.e., action potential–independent glutamate release) was assessed. As shown previously (14), AP5 had no effect on mEPSC frequency, amplitude, or charge transfer in 6/6 neurons from 3 control rats. Specifically, in the presence of AP5, mEPSC frequency, amplitude, and charge transfer were 94% ± 4.9%, 93.6% ± 2.10%, and 91.2% ± 2.77% of baseline, respectively (*P >* 0.05 for each). Similarly, memantine itself had no effect upon mEPSC frequency, amplitude, or charge transfer in 8/11 neurons from 5 rats (104% ± 15.8%, 94.7% ± 3.8%, 100% ± 14.0%, respectively; *P >* 0.05 for each). Subsequent application of AP5 in the presence of memantine had no effect on mEPSC frequency (117.4% ± 33.77%), amplitude (94.1% ± 3.05%), or charge transfer (153.7% ± 54.05%; *P >* 0.05 for each; Supplemental Table 2 and Figure 2, A and C).

To assess whether synaptic NMDAR–dependent currents could be uncovered under control conditions, the effects of the glutamate uptake inhibitor, DHK, were assessed. Application of DHK had no significant effect on mEPSC frequency per se in 12/14 control DMV neurons (99% ± 44% of baseline; *P >* 0.05; *n =* 14 neurons, 4 rats), but uncovered AP5-sensitive, synaptic NMDAR–mediated currents in 9/13 neurons tested (mEPSC frequency 77% ± 6.2%; charge transfer 77% ± 9.0%; *P <* 0.05 for both), but had no effect on mEPSC amplitude (93% ± 3.79%; *P >* 0.05; Supplemental Table 2 and Figure 2, A and C). Although alterations in mEPSC frequency are normally considered to indicate a presynaptic site of action, previous studies (14) determined this is likely due to a threshold effect, where mEPSC amplitude is reduced below the range of detection, suggesting the observed effects of the antagonist do indeed occur at postsynaptic sites of action.

### aHFD exposure activates synaptic NMDARs subsequent to extrasynaptic NMDARs in DMV neurons.

In contrast to the observations in control DMV neurons, after aHFD exposure, application of DNQX (30 μM) reduced the amplitude of eEPSCs to a significantly lesser extent (from 141 ± 13.0 pA to 112 ± 10.1 pA; *P <* 0.05 versus controls; *n =* 6), confirming that non–AMPA-mediated currents contributed significantly to glutamatergic transmission, even at negative membrane potentials (14). The remaining AMPA-insensitive evoked current was reduced significantly by the extrasynaptic NMDAR antagonist, memantine (30 μM; 25 ± 5.9 pA; *P <* 0.05 versus DNQX alone), which recovered upon washout (90 ± 26.6 pA), suggesting that in aHFD conditions, extrasynaptic NMDARs are activated endogenously (Figure 1, C and D).

As shown previously (14), AP5 decreased mEPSC frequency in 6/6 aHFD DMV neurons from 3 rats. Specifically, in the presence of AP5, mEPSC frequency and charge transfer were decreased (64% ± 8.2% and 67% ± 6.7% of baseline, respectively; *P <* 0.05), whereas mEPSC amplitude was unaffected (109% ± 4.0%; *P >* 0.05; Supplemental Table 2 and Figure 2, B and D). To ascertain whether these NMDA-mediated currents were dependent upon the constitutive activation of extrasynaptic NMDARs, the ability of memantine to block synaptic NMDAR events was assessed. Although memantine itself had no significant effect on mEPSC frequency, amplitude, or charge transfer in 5/6 DMV neurons from 3 rats (87% ± 8.6%, 89% ± 5.08%, and 81% ± 2.2%, respectively; *P >* 0.05 for each), it blocked the actions of subsequent application of AP5 to inhibit glutamatergic transmission (frequency: 96% ± 6.2%; amplitude: 103% ± 3.09%; charge transfer: 107% ± 14.5% of memantine; *P >* 0.05 for each; Supplemental Table 2 and Figure 2, B and D).

An additional series of experiments was conducted in 6 aHFD DMV neurons from 3 rats in which the effects of the synaptic NMDAR antagonist, MK801 (5 μM), were assessed. In all 6 neurons tested, MK801 decreased mEPSC frequency and charge transfer (59% ± 5.2% and 63% ± 5.7%, respectively; *P <* 0.05) without affecting amplitude (92% ± 2.9%; *P >* 0.05; Supplemental Table 2), confirming that aHFD induced synaptic NMDAR activation. Subsequent application of memantine in the continued presence of MK801 had no further effect on mEPSC frequency, charge transfer, or amplitude (106% ± 3.1%, 99% ± 4.5%, 93% ± 2.9%, respectively; *P >* 0.05 for each; Supplemental Table 2), confirming that activation of synaptic NMDARs is dependent upon activation of extrasynaptic NMDARs.

To determine whether extrasynaptic NMDARs were activated maximally after the aHFD, mEPSCs were recorded in the presence of DHK, which itself had no effect on mEPSC frequency, charge transfer, or amplitude (90% ± 10.1%, 88% ± 12.0%, 86% ± 2.3% of baseline, respectively; *n =* 11 neurons, 3 rats). Subsequent addition of AP5 decreased mEPSC frequency and charge transfer (61% ± 7.4% and 47% ± 6.9%; *P <* 0.05 versus DHK) as before (14), with no effect on mEPSC amplitude (83% ± 4.0%; *P >* 0.05 Supplemental Table 2 and Figure 2, B and D). Of note, the magnitude of the AP5-mediated decrease in mEPSC frequency was similar to that uncovered in control DMV neurons after application of DHK (*P >* 0.05 versus control neurons; Supplemental Table 2 and Figure 2, A and C), suggesting that extrasynaptic NMDARs are, indeed, activated maximally in response to aHFD exposure.

To further confirm the involvement of extrasynaptic NMDARs, the effects of ifenprodil on mEPSC properties were assessed. Ifenprodil had no effect on mEPSC frequency, amplitude, or charge transfer in 5/5 control neurons from 3 rats (95% ± 5.2%, 101% ± 3.5%, and 107% ± 6.9%, respectively; *P >* 0.05 for each). In contrast, in 6/6 aHFD neurons from 3 rats, ifenprodil decreased mEPSC frequency and charge transfer (70% ± 1.4% and 70.3% ± 6.1%; *P <* 0.05) with no effect on mEPSC amplitude (107% ± 5.5%; *P >* 0.05; Supplemental Table 2 and Supplemental Figure 2). In 5 of these 6 neurons tested further, the actions of AP5 to decrease mEPSC frequency and charge transfer were abolished in the presence of ifenprodil (92.6% ± 3.7% and 107.4% ± 15.1%; *P >* 0.05) with no effect on amplitude (92% ± 3.7%; *P >* 0.05; Supplemental Table 2 and Supplemental Figure 2).

In a further 7 aHFD DMV neurons from 3 rats, the effects of the selective NR2B antagonist, Conantokin-G (ConG; refs. 41, 42) were assessed. Perfusion with ConG (0.6 μM) itself decreased mEPSC frequency and charge transfer (60% ± 3.2% and 64% ± 3.6%; *P <* 0.05) but not amplitude (101% ± 3.2%; *P >* 0.05). Subsequent application of AP5 had no effect on mEPSCs (frequency: 90% ± 7.7%; charge transfer 94% ± 8.1%; amplitude 96% ± 2.5%; *P >* 0.05 for each; Supplemental Table 2).

Collectively, these data suggest that the uncovering of synaptic NMDAR–mediated responses after aHFD was dependent upon the activation of extrasynaptic NMDARs, and that aHFD induced maximal activation of extrasynaptic NMDARs. Furthermore, although silent under normal diet conditions, a similar mechanism could be uncovered in control DMV neurons if glutamate levels are increased.

### aHFD-induced modulation of DMV neuronal excitability is dependent upon activation of the extrasynaptic NMDARs.

To verify that the aHFD-induced activation of extrasynaptic NMDARs had a physiologically relevant effect on DMV neuronal excitability, additional experiments were conducted in which the effects of extrasynaptic NMDAR and synaptic NMDAR antagonists on holding current, cell-attached firing rate, and action potential firing frequency were assessed.

In neurons voltage-clamped at –50 mV, AP5 (25 μM) had no effect on holding current in either control (–0.6 ± 0.92 pA) or aHFD neurons (7.1 ± 1.6 pA; *n =* 5 neurons, 3 rats for each; *P >* 0.05 for each). In contrast, although memantine had no effect on holding current in control neurons (–1.4 ± 1.92 pA, *n =* 21 neurons, 8 rats; *P >* 0.05), it induced an outward current in aHFD neurons (17.6 ± 3.3 pA; *n =* 13 neurons, 5 rats; *P <* 0.05); notably, the memantine-induced outward current was of similar magnitude even after perfusion with the synaptic NMDAR antagonist, MK801 (16.7 ± 2.74 pA; *n =* 7 neurons, 3 rats). Similarly, ifenprodil had no effect on holding current in control DMV neurons (0.8 ± 2.1 pA; *n =* 9 neurons, 4 rats), whereas DHK induced an inward current (10.0 ± 1.3 pA; *n =* 13 neurons, 4 rats; *P <* 0.05). In contrast, both ifenprodil and ConG induced outward currents in aHFD neurons (16.1 ± 3.76 pA; *n =* 11 neurons, 6 rats and 12.3 ± 2.24 pA; *n =* 9 neurons, 3 rats; *P <* 0.05 for each), whereas DHK had no effect (3.7 ± 1.07 pA; *n =* 11 neurons, 6 rats; *P >* 0.05; Supplemental Figure 3).

To determine whether the aHFD-induced increase in synaptic NMDAR activation influenced the basal firing rate of DMV neurons, the cell-attached firing rate was assessed in 20 control neurons from 6 rats and 23 aHFD neurons from 10 rats. The cell-attached firing rate was not different between the 2 groups of neurons (1.3 ± 0.15 versus 1.6 ± 0.19 events.s^–1^; *P >* 0.05; Supplemental Figure 4), suggesting that despite the upregulation in glutamatergic signaling, the aHFD did not increase the spontaneous firing frequency of DMV neurons.

**Figure 3:**
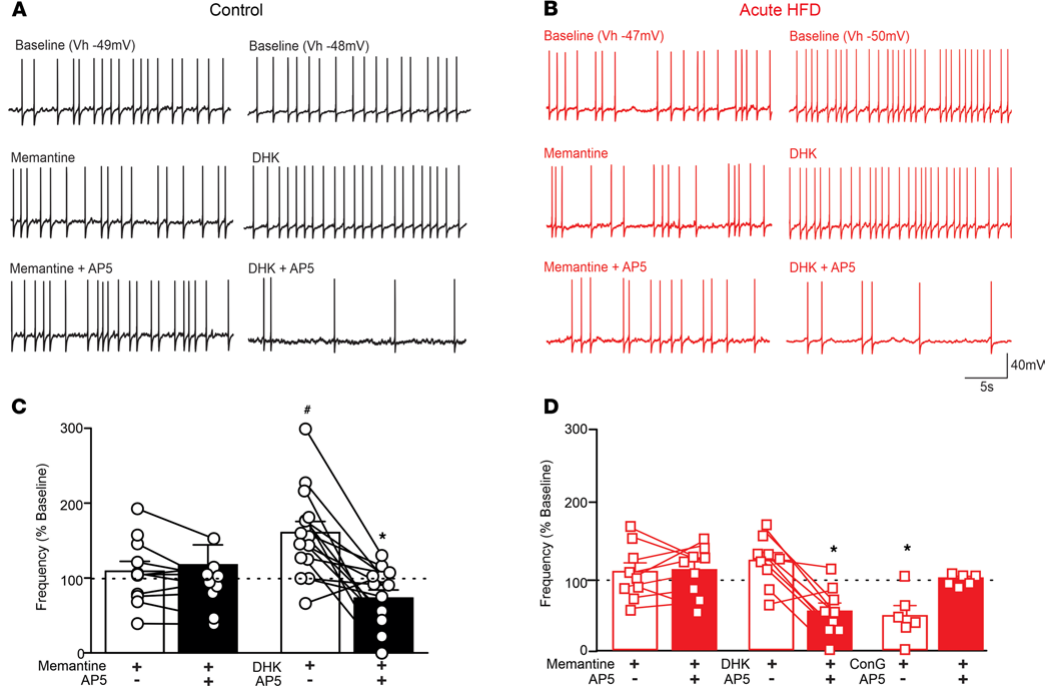
Extrasynaptic NMDAR activation is required for the synaptic NMDAR–mediated decrease in action potential firing rate. (**A** and **B**) Representative traces from gastric-projecting control (**A**) and aHFD (**B**) DMV neurons current-clamped at a potential to allow action potential firing at approximately 1 Hz. In controls (**A**), perfusion with memantine (30 μM; left, middle) or AP5 (25 μM; left, bottom) had no effect on action potential firing rate. Perfusion with DHK, however, increased action potential firing rate (30 μM; right, middle) and uncovered an AP5-mediated decrease in action potential firing rate (right, bottom). In aHFD neurons (**B**), perfusion with memantine (30 μM; left, middle) had no effect on action potential firing rate but blocked the subsequent AP5-mediated decrease (25 μM; left, bottom) and perfusion with DHK (30 μM; right, middle) had no effect on action potential firing rate and did not affect the observed AP5-mediated decrease (right, bottom). (**C** and **D**) Graphical summary of the effects of memantine (**C**; left, *n =* 11 cells, 5 rats) (**D**; left, *n =* 9 cells, 4 rats), DHK (**C**; right; *n =* 16 cells, 6 rats) (**D**; middle; *n =* 9 cells, 3 rats), and AP5 on action potential firing rate in control (**C**) and aHFD (**D**) DMV neurons. In controls (**C**), application of memantine and AP5 had no effect on action potential firing rate. Application of DHK, however, significantly increased action potential firing rate in control conditions, which uncovered an AP5-mediated decrease in action potential firing rate. After aHFD exposure (**D**), application of memantine (left) and ConG (right; *n =* 6 cells, 3 rats) attenuated the AP5-mediated decrease in action potential firing rate observed after aHFD exposure. Application of DHK had no significant effect on action potential firing rate and did not affect the AP5-mediated decrease observed normally. *P < 0.05 versus DHK ^#^*P <* 0.05 versus baseline (Student’s paired *t* test).

In control DMV neurons, neither memantine nor the subsequent application of AP5 had any effect on action potential firing rate (0.6 ± 0.04, 0.6 ± 0.1, and 0.6 ± 0.10 events.s^–1^, respectively; *P >* 0.05; *n =* 11 neurons, 4 rats; Figure 3, A and C). To ascertain whether the exogenous activation of extrasynaptic NMDARs affected neuronal excitability, the ability of DHK to uncover AP5-mediated effects was assessed. DHK itself increased action potential firing rate (1.00 ± 0.17 versus 0.5 ± 0.06 events.s^–1^; *P <* 0.05, *n =* 16 neurons, 6 rats), and subsequent application of AP5 decreased the action potential firing rate (0.7 ± 0.13 events.s^–1^; *P <* 0.05; Figure 3, A and C).

In contrast, in aHFD conditions, although memantine itself had no effect on action potential firing rate (0.6 ± 0.08 versus 0.6 ± 0.06 events.s^–1^; *P >* 0.05; *n =* 9 neurons, 4 rats), it blocked the ability of AP5 to decrease neuronal excitability (0.6 ± 0.07 events.s^–1^; *P >* 0.05). Additionally, although DHK had no effect on action potential firing rate in aHFD conditions (0.7 ± 0.08 versus 0.5 ± 0.07 events.s^–1^; *P >* 0.05; *n =* 9 neurons, 3 rats), subsequent application of AP5 decreased the action potential firing rate (0.3 ± 0.09 events.s^–1^; *P <* 0.05 versus DHK; Figure 3, B and D) to an extent similar to that observed in control neurons (*P >* 0.05 versus control), again suggesting that extrasynaptic NMDARs were activated maximally after the aHFD. An additional series of experiments were conducted in 6 aHFD DMV neurons from 3 rats in which the ability of the NR2B-selective antagonist, ConG, to alter action potential firing rate was assessed. ConG itself decreased action potential firing rate in all 6 neurons tested (0.4 ± 0.14 versus 0.8 ± 0.05 events.s^–1^; *P <* 0.05), whereas subsequent application of AP5 had no further effect on action potential firing rate (0.92 ± 0.02 versus 0.93 ± 0.03 events.s^–1^; Figure 3D), again confirming the involvement of extrasynaptic NMDARs in the induction of synaptic NMDAR activation.

**Figure 4:**
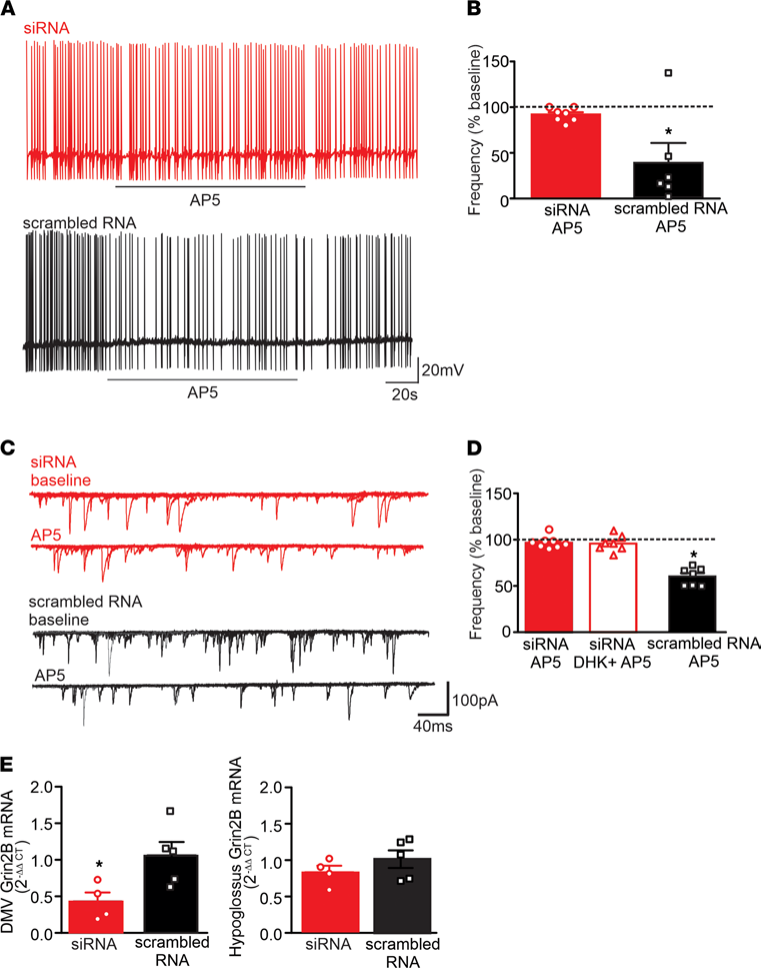
siRNA-mediated knockdown of GRIN2B prevents the activation of synaptic NMDARs. (**A**) Representative traces from gastric-projecting aHFD DMV neurons after microinjection of siRNA targeted against GRIN2B (red, upper) or scrambled RNA controls (black, lower). Neurons were current-clamped at a potential that allowed action potential firing of approximately 1 Hz. Perfusion with AP5 (25 μM) had no effect on action potential firing rate in siRNA rats, but decreased action potential firing rate in scrambled RNA rats. (**B**) Graphical summary of the effects of AP5 on action potential firing GRIN2B siRNA rats (red; *n =* 7 cells, 3 rats) and scrambled RNA rats (black; *n =* 6 cells, 3 rats) after aHFD exposure. Neurons were current-clamped at a potential that allowed for action potential firing of approximately 1 Hz. AP5 had no effect on action potential firing rate in siRNA rats (red; left), but decreased action potential firing rate in scrambled RNA rats. *P < 0.05 versus baseline (Student’s paired *t* test). (**C**) Six overlapping consecutive traces from gastric-projecting aHFD DMV neurons voltage-clamped at –50mV illustrating mEPSCs in siRNA (red, upper) or scrambled RNA (black, lower) microinjected rats. Perfusion with AP5 (25 μM) decreased mEPSC frequency in scrambled but not siRNA rats. (**D**) Graphical summary of the effects of AP5 on mEPSC frequency in siRNA and scrambled RNA rats. AP5 had no effect on mEPSC frequency in siRNA rats (red, left), even after perfusion with DHK (red open, middle). Conversely, AP5 decreased mEPSC frequency in scrambled RNA rats (black, right). *P < 0.05 versus baseline (1-way ANOVA followed by post hoc Dunnett’s multiple-comparison test). (**E**) Graphical summary of GRIN2B gene expression measured by qPCR in the DMV (left) and hypoglossus (right; control region) in aHFD rats microinjected with siRNA (*n =* 4 rats) or scrambled RNA (*n =* 5 rats). Each experiment had 2 replicates. The siRNA injection reduced GRIN2B mRNA by approximately 60% in the DMV with no effect in hypoglossus. Data were normalized to β-actin and expressed as fold change using the 2^–ΔΔCT^ method. *P < 0.05 versus scrambled RNA (Student’s unpaired *t* test).

A final series of experiments was conducted to assess whether siRNA-mediated knockdown of the extrasynaptic NMDAR NR2B subunit attenuated the aHFD-induced upregulation of synaptic NMDAR–mediated currents. In 7 neurons from 3 aHFD rats in which the DMV was injected with siRNA directed against the NMDAR NR2B subunit, perfusion with AP5 had no effect on action potential firing rate (0.7 ± 0.09 versus 0.8 ± 0.11 events.s^–1^; *P >* 0.05), whereas AP5 decreased the firing rate in 5/6 neurons from 3 rats injected previously with scrambled RNA (0.2 ± 0.11 versus 0.8 ± 0.12 events.s^–1^; *P <* 0.05; Figure 4, A and B). Similarly, although AP5 decreased mEPSC frequency and charge transfer (60% ± 3.6% and 64% ± 3.8%, respectively; *P <* 0.05) without affecting amplitude (102% ± 3.5%; *P >* 0.05) in 7 neurons from 4 rats injected with scrambled RNA, it had no effect on mEPSC frequency, charge transfer, or amplitude in 8 neurons from 3 siRNA-injected rats (97% ± 2.3%, 101% ± 7.1%, and 103% ± 2.7%, respectively; *P >* 0.05; Supplemental Table 2 and Figure 4, C and D). Of note, in 7 of those aHFD DMV neurons, perfusion with DHK did not uncover any AP5-sensitive, synaptic NMDAR–mediated effects (frequency 96% ± 3.2%, charge transfer 100% ± 2.6%, amplitude 99% ± 2.0%; *P >* 0.05 for each; Supplemental Table 2). The efficacy of the siRNA-mediated knockdown was confirmed by measurement of gene expression of the GRIN2B subunit in micropunches of DMV in siRNA-injected rats (*n =* 5) and scrambled RNA–injected rats (*n =* 6), with the adjacent hypoglossus used as control tissue. Notably, the results showed an approximately 60% decrease in GRIN2B mRNA in DMV after siRNA injection, with no difference in GRIN2B gene expression in hypoglossal nucleus between scrambled RNA– and siRNA-injected rats (Figure 4E).

### aHFD-induced modulation of gastric tone and motility is dependent upon activation of the extrasynaptic NMDARs.

To investigate whether the aHFD-mediated alterations in glutamatergic synaptic events and DMV excitability have any physiologically relevant actions on vagally dependent efferent control of gastric functions, experiments were conducted in which the effects of brainstem application of ionotropic glutamate antagonists on gastric motility and tone were assessed.

**Figure 5:**
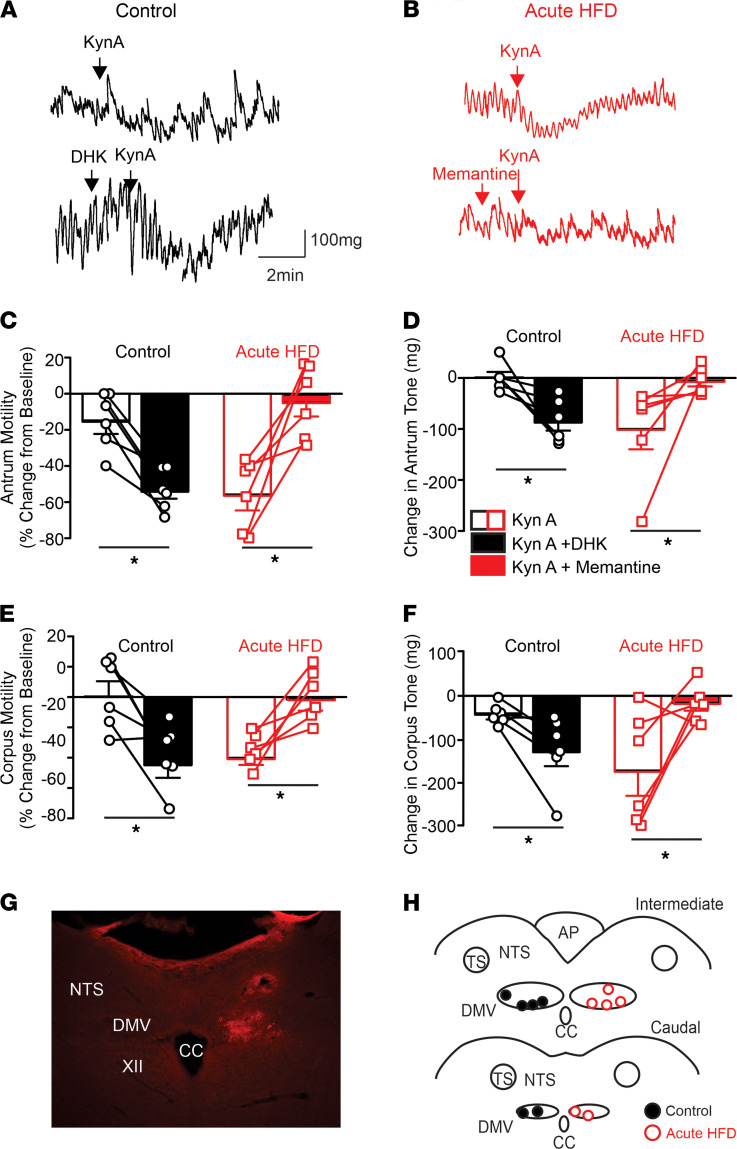
The synaptic NMDAR–mediated decrease in gastric motility and tone observed after aHFD exposure is dependent upon activation of extrasynaptic NMDARs. (**A** and **B**) Representative gastric motility traces from control (**A**) and aHFD (**B**) rats. In control conditions (**A**), DVC microinjection of the nonselective glutamate receptor antagonist, kynurenic acid (KynA; 100 pmol/60 nL) had no effect on antrum tone and motility (upper trace). In contrast, DVC microinjection of KynA after fourth ventricular application of the glutamate reuptake inhibitor, dihydrokinate (DHK; 1 mM in 2 μL; lower trace) decreased gastric tone and motility. After aHFD (**B**), DVC microinjection KynA; (100 pmol/60 mL) decreased gastric tone and motility (upper trace). In contrast, DVC microinjection of KynA after fourth ventricular application of memantine (50 mM in 2 μL; lower trace) had no significant effect on gastric tone or motility. (**C**–**F**) Graphical representation of the effects of brainstem microinjection of KynA, DHK, and memantine on antrum (**C** and **D**) and corpus (**E** and **F**) motility (**C** and **E**) and tone (**D** and **F**) in control (left; *n =* 6) and aHFD (right; *n =* 6) rats. (**G**) Photomicrograph illustrating a brainstem microinjection (arrow) in the intermediate DVC. XII = hypoglossus; NTS = nucleus of the tractus solitarius; DMV = dorsal motor nucleus of the vagus; CC = central canal. (**H**) Map illustrating all brainstem microinjection sites, divided into intermediate (top) and caudal (lower) areas. For the sake of clarity, injections are marked bilaterally (control; left, HFD; right), although all microinjections were made into the left DVC since recordings of motility and tone were made from the ventral stomach. *P < 0.05 versus KynA alone (Student’s paired *t* test).

In vivo recordings of gastric tone and motility were made in response to dorsal vagal complex (DVC) microinjection of the nonselective ionotropic glutamate antagonist, kynurenic acid (KynA; 100 pmol/60 nL) before and after fourth ventricular application of memantine (aHFD rats; 50 pmol in 2 μL) or DHK (control rats; 1 mM in 2 μL). As demonstrated previously (14), after aHFD exposure, brainstem microinjection of KynA decreased the motility of the antrum and the corpus in 6/6 and 5/6 rats, respectively. Fourth ventricular application of memantine, however, attenuated or abolished these gastroinhibitory actions in all 6 rats (Table 1 and Figure 5). In contrast, in control conditions where brainstem injection of KynA had no effect in 5/6 rats (14), fourth ventricular application of DHK uncovered the ability of KynA to decrease gastric tone and motility (Table 1 and Figure 5, A, C, and E).

These data suggest that the synaptic NMDAR–dependent increase in vagally mediated gastric motility and tone observed after the aHFD was a centrally mediated response that was dependent upon extrasynaptic NMDAR activation.

### aHFD exposure significantly delays gastric emptying but does not alter gastric contractility.

**Figure 6:**
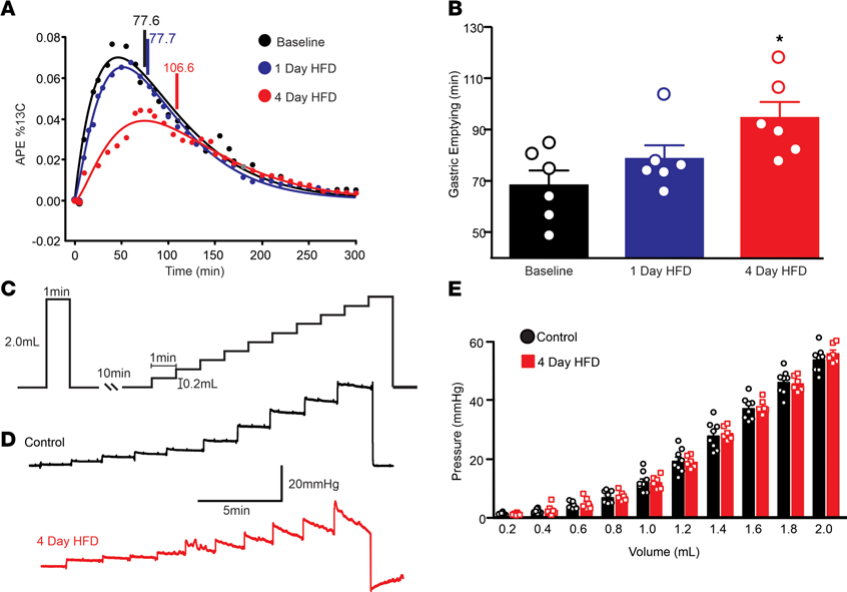
aHFD exposure significantly delays gastric emptying and does not affect compliance. (**A**) Representative gastric emptying curves in atomic percentage excess (APE; %13C). Half emptying times (T_1/2_) are indicated by vertical bars. T_1/2_ was significantly delayed after 4 days of HFD exposure (red) compared with baseline (black) and 1 day of HFD exposure (blue). (**B**) Graphical summary of gastric emptying (T_1/2_; min) in rats (*n =* 6) throughout exposure to HFD. Gastric emptying was significantly delayed after 4 days of HFD exposure (red) compared with baseline (black) and 1 day of HFD exposure (blue). *P > 0.05; 1-way ANOVA followed by post hoc Dunnett’s multiple-comparison test. (**C**) Schematic diagram illustrating the balloon inflation protocol for gastric compliance experiments. (**D**) Representative sample traces of gastric compliance in control (black; top) and aHFD (red; bottom) rats. There is no significant difference in compliance between control and aHFD traces. (**E**) Graphical summary of balloon pressure in response to increased volume in control (black) and aHFD (red) rats. There is no significant difference between control and 4-day HFD at any volume. *P > 0.05 (2-way ANOVA).

The ^13^C octanoic acid breath test was used to assess whether aHFD exposure alters gastric emptying in male rats (*n =* 6). When tested after 4 days of HFD exposure, gastric emptying was delayed significantly compared with both baseline and 1-day HFD exposure (when caloric intake was dysregulated) (T_1/2_: 96 ± 5.4 minutes versus 68 ± 5.8 minutes versus 78 ± 5.4 minutes, respectively; *P <* 0.05 compared with 1-day HFD and baseline, Figure 6, A and B).

To investigate whether aHFD exposure induced any peripheral changes in gastric function, gastric compliance was measured in control (*n =* 6) and aHFD (*n =* 8) rats. After 4 days of HFD exposure, however, gastric compliance was not different from that of control rats at any volume tested (Figure 6, C–E).

These data suggest that the alterations in gastric motility and tone observed after aHFD occurred via central, not peripheral, neuromodulation.

### The homeostatic regulation of caloric intake after aHFD exposure is dependent upon activation of the extrasynaptic NMDARs.

To investigate whether the upregulation in DMV NMDAR-mediated glutamatergic signaling is responsible for the homeostatic regulation of caloric intake observed after the aHFD, food intake was assessed in response to chronic brainstem application of memantine.

**Figure 7:**
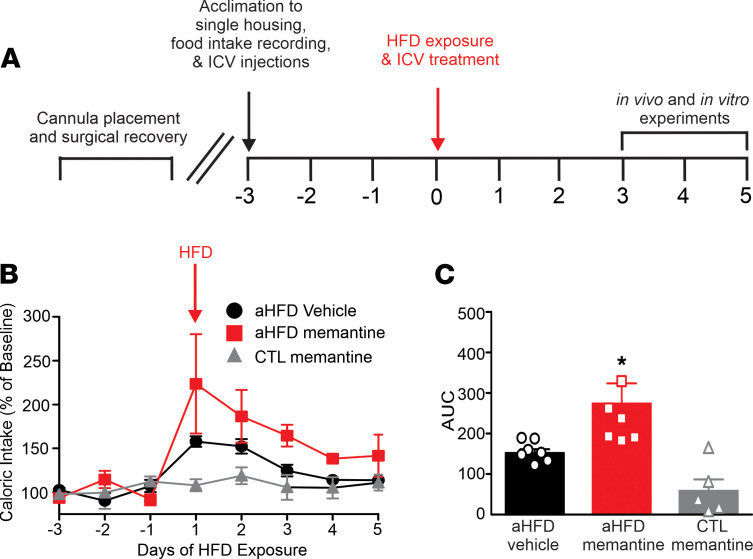
Homeostatic regulation of caloric intake during aHFD exposure is attenuated by chronic fourth ventricular application of memantine. (**A**) Schematic diagram of the experimental timeline. (**B**) Graphical summary of caloric intake after exposure to aHFD. Note that fourth ventricular application of memantine (*n =* 6 rats) attenuated the homeostatic regulation of caloric intake observed in vehicle-treated animals (*n =* 7 rats) upon exposure to HFD; memantine had no effect on caloric intake in control rats (*n =* 5 rats). (**C**) Graphical summary of caloric intake represented as AUC. HFD rats treated with chronic fourth ventricular memantine (*n =* 6 rats) consumed significantly more than vehicle treated (*n =* 7 rats) and control (*n =* 5) rats. *P < 0.05 versus vehicle and ^#^*P <* 0.05 versus aHFD (1-way ANOVA followed by post hoc Dunnett’s multiple-comparison test).

After surgical implantation of chronic indwelling fourth ventricular cannulae, the effects of chronic brainstem application of memantine (50 pmol in 2 μL; *n =* 6) or vehicle (PBS; 50 pmol in 2 μL; *n =* 7) to modulate food intake in response to an HFD were assessed (Figure 7A). Compared with vehicle administration, memantine attenuated the homeostatic regulation of caloric intake observed after aHFD exposure (AUC = 152 ± 9.6 versus 274 ± 49.7 kcal; *P <* 0.05; Figure 7, B and C). In a further 5 rats maintained on a control diet throughout, memantine itself had no effect on caloric intake (AUC = 58 ± 28.6 kcal; *P <* 0.05 versus aHFD vehicle and aHFD memantine; Figure 7, B and C). These data confirmed that extrasynaptic NMDARs did not play any role in caloric intake under control conditions and suggest that the restoration of caloric balance observed 3–5 days after HFD exposure was dependent upon the activation of extrasynaptic NMDARs and subsequent activation of synaptic NMDARs within the DVC.

## Discussion

The results from the present study suggest that after aHFD exposure, activation of extrasynaptic NMDARs 1) is required for the activation of synaptic NMDAR–dependent currents and the preservation of gastric-projecting DMV neuronal excitability; 2) is responsible for the synaptic NMDAR modulation of vagally dependent gastric tone, motility, and emptying; and 3) results in the homeostatic regulation of caloric intake.

The DVC functions as a critical intersection in the integration of ascending interoceptive signals and descending visceromotor signals related to food intake, satiation, and energy balance. Multiple studies have described disruption and dysregulation of vago-vagal neurocircuits in response to HFD and after the development of diet-induced obesity, with decreased excitability and responsiveness of both vagal afferent (sensory) and efferent (motor) have been reported (8–13). A smaller number of studies have shown, however, that neuroplasticity also occurs within vagal neurocircuits after relatively short periods of HFD exposure (13, 14). Notably, studies in human and animal models have shown that a brief (24 hour) period of hyperphagia occurs in response to HFD exposure, which is followed by a decrease in food intake and the restoration of caloric balance within 3 to 5 days (14–17). This period of caloric regulation is associated with upregulation of vagally dependent glutamatergic signaling within the brainstem (14), but until now, it was unclear whether the observed neuroplasticity was required for or simply associated with the homeostatic regulation of energy balance. The current study provides a mechanistic basis for understanding the neuroplasticity responsible for this regulation and the speculation that failure to engage this mechanism appropriately, or loss of this plasticity after prolonged HFD exposure, may lead to caloric imbalance, weight gain, and obesity.

Because of their unique biophysical membrane properties and complement of voltage-dependent currents, DMV neurons are spontaneously active pacemaker cells (23, 43). The activity and excitability of DMV neurons are also significantly affected by GABAergic, glutamatergic, and catecholaminergic synaptic inputs from the adjacent NTS (23, 44, 45). In vivo and in vitro studies have shown that under physiological conditions, inhibitory GABAergic inputs are the principal regulators of DMV neuronal activity and vagal efferent control of gastric functions, whereas excitatory glutamatergic projections do not appear to play a vital role in regulating gastric functions under basal conditions, although evidence suggests they may be more involved in pancreatic functions (23, 46, 47). However, glutamatergic synapses may play a more significant role in the adaptation of vagal neurocircuitry in response to pathophysiological conditions. Hindbrain NMDA receptors are known to play critical roles in the termination of food intake and the regulation of high sucrose intake (19, 42), and we have shown previously that aHFD exposure is associated with the activation of synaptic NMDARs on gastric-projecting DMV neurons. The temporal pattern of this glutamatergic neuroplasticity corresponds to the period of homeostatic regulation of caloric intake, suggesting an association between the observed responses (14). The current study confirmed and extended these observations, while demonstrating that synaptic NMDAR activation was not only necessary for the restoration of caloric balance, but the upregulation of their signaling occurred in response to activation of extrasynaptic NMDARs. Of note, the basal action potential firing rate was similar between control and aHFD DMV neurons. Together with our findings that memantine, ifenprodil, and ConG all induced significant outward currents in aHFD neurons, this suggests that aHFD-dependent activation of extrasynaptic NMDARs increased excitatory glutamatergic signaling to restore DMV neuronal activity and caloric balance.

Food intake and meal patterning are determined by many factors, including gastric emptying, compliance, and motility patterns (48). The present study did not find any significant difference in gastric compliance after the aHFD, suggesting the regulation of caloric intake did not involve alterations in the neuromuscular function of the stomach. The restoration of caloric balance was associated with a delay in gastric emptying, however. Although no changes in basal action potential firing rate were observed between control and aHFD neurons in single-unit electrophysiological recordings, the delayed gastric emptying observed at this time point suggests that a loss of coordination and synchronicity in cumulative vagal efferent activity may be involved in dysregulated and inefficient gastric contractility patterns, resulting in delayed gastric emptying and the regulation of caloric intake (48). The prominent role of NMDA receptors in cardiorespiratory functions (49–52) and the sensitivity of the ^13^C octanoic breath test to changes in respiration rate presented a significant confounder to investigations into the role of NMDARs in altered gastric emptying, and therefore prohibited further experimentation. Nevertheless, chronic inhibition of brainstem extrasynaptic NMDARs attenuated the homeostatic regulation of caloric intake, implying that their activation plays a central role in this physiological compensation.

Under basal conditions, glutamatergic NTS-DMV signaling involves principally non-NMDA (presumably AMPA) receptor activation, due to the voltage-dependent Mg^2+^ block of NMDA receptors at hyperpolarized membrane potentials (14, 23, 24). Synaptic NMDARs are activated constitutively after aHFD exposure, even at membrane potentials that would not normally suggest depolarization-induced removal of the Mg^2+^ ion channel block (14, 24). Although it is certainly possible that a lack of appropriate space control may allow for a sufficient membrane depolarization to remove the Mg^2+^ blockade, previous studies have shown that the dendritic structure of DMV neurons does not affect voltage behavior significantly (53) and more prolonged HFD exposure does not contribute to space clamp problems (11). Although we cannot discount the possibility that aHFD exposure alters the Mg^2+^ blockade of NMDAR, the present study suggests that, as shown in other central areas (25, 26), activation of DMV extrasynaptic NMDARs allows the activation of synaptic NMDARs. Importantly, the current study also demonstrated that the same mechanism exists yet is silent under basal (control) conditions, but can be uncovered if synaptic glutamate levels are elevated. Even under physiological conditions, therefore, central vagal neurons display several mechanisms by which synaptic efficacy can be modulated rapidly, further reinforcing the extant literature supporting the remarkable degree of plasticity within these neurocircuits (6, 7, 54, 55).

It should also be noted, however, that similar to their synaptic counterparts, extrasynaptic NMDARs may also display a voltage-dependent Mg^2+^ block. Although the extent of this block may be dependent upon the receptor subunit composition (27–29), it is also possible that some additional mechanism by which DMV neuronal excitability is modulated may also be involved. Given that the observed plasticity occurs in glutamatergic synapses, the most metabolically economical means for removal of the Mg^2+^-dependent block of extrasynaptic NMDARs and synaptic NMDARs may involve the activation of postsynaptic metabotropic glutamate receptors (56, 57), which are distributed throughout the vagal brainstem (58–64). Even if this were the case, though, the source of such glutamate may not necessarily be synaptic, particularly since there is no evidence supporting an increase in the amount of glutamate released after aHFD (14). Of note, however, astroglial activation occurs in hypothalamic regions after similar short periods of HFD exposure (65–68), and alterations in either the astroglial uptake of synaptic glutamate or the release of gliotransmitters (including glutamate) appears to be an increasingly important mechanism by which synaptic efficacy can be modulated rapidly (25, 26, 69, 70). It should also be noted, however, that the release of additional gliotransmitters to activate, for example, postsynaptic purinergic (71) or TRPV1 receptors (72), should also be considered as a viable means by which subsequent activation of extrasynaptic NMDARs and synaptic NMDARs may occur.

The results from the present study suggest that the activation of DMV synaptic NMDARs observed after the aHFD is dependent upon activation of extrasynaptic NMDARs. As pacemakers, DMV neurons have a resting membrane potential within 1–2 mV of action potential firing threshold (23, 43), and even small alterations in neuronal excitability can have profound effects on efferent output, hence gastric functions. The observed neuroplasticity within glutamatergic synaptic transmission, the increase in vagally dependent efferent drive to the stomach, and the delay in gastric emptying appear responsible for the regulation of caloric intake after aHFD exposure. Although the exact means by which extrasynaptic NMDARs become activated remains to be elucidated, this glutamatergic neuroplasticity is critical to understanding caloric regulation, and importantly, may provide crucial information related to how and why energy balance becomes dysregulated during chronic HFD exposure.

## Methods

### Animals.

Male and virgin female Sprague-Dawley (SD) rats (*n =* 128 total; 77 males, 51 females; Charles River Laboratories) were used for electrophysiological (*n =* 66; 31 males, 35 females) and food intake studies (*n =* 18; 10 females, 8 males), and quantitative PCR (qPCR) measurement of GRIN2B subunit mRNA (*n =* 6 females; 5 males). Only male SD rats were used for in vivo gastric motility (*n =* 12), compliance (*n =* 14), and emptying (*n =* 6) recordings because of variability in gastric functions in females throughout the estrus cycle (73). All rats were housed in Penn State College of Medicine’s temperature-controlled (20°C–26°C) and humidity-controlled (30%–70%) Central Animal Quarters with an artificial 12-hour light/12-hour dark cycle. All rats were group-housed in plastic, open-top cages with wire lids and corn cob bedding, with ad libitum access to water and normal chow (fat, protein, carbohydrate content 14%:27%:59%; Purina Mills) or an HFD (60%:20%:20%, D12492; Research Diets), which was provided for 3 to 5 days prior to experimentation, unless otherwise stated. All rats were 4 to 8 weeks of age during in vitro and in vivo experiments.

### Identification of gastric-projecting DMV neurons.

Prior to electrophysiological experiments, gastric-projecting DMV neurons of rats were identified through application of the retrograde tracer, DiI (octadecyl [C18] indocarbocyanine; Life Technologies) to either the corpus or the antrum of the stomach, as described previously (74). Briefly, rats were anesthetized with isoflurane (2.5% in 100% O_2_) to a deep plane of anesthesia and the foot pinch reflex was lost. An abdominal laparotomy exposed the stomach, and DiI was applied to either the corpus or the antrum/pylorus and secured in place with epoxy resin. The rats were allowed a 2-week recovery period, which is sufficient time for the tracer to reach the gastric-projecting DMV neurons.

### Brainstem preparation.

Two weeks after the labeling of gastric-projecting DMV neurons, rats (*n =* 66, 35 females, 31 males) were anesthetized with isoflurane before euthanasia via bilateral pneumothorax. The brainstem was removed and sliced as described previously (74, 75). Briefly, the brainstems were submerged in cold (~5°C) oxygenated Krebs solution (in mM: 126 NaCl, 25 NaHCO_3_, 2.5 KCl, 1.2 MgCl_2_, 2.4 CaCl_2_, 1.2 NaH_2_PO_4_, and 10 D-glucose, maintained at pH 7.4 by bubbling with 95% O_2_/5% CO_2_). The brainstems were then mounted on a vibratome, sliced into 300 μm sections, and placed in warm (30°C) oxygenated Krebs solution for at least 90 minutes before recording.

### Electrophysiological recordings from brainstem slices.

Brainstem slices were individually placed in a perfusion chamber (volume 500 μL) fitted on the stage of a Nikon E600FN microscope equipped with TRITC epifluorescent filters. Slices were perfused at a rate of 2.0 to 2.5 mL/min with warmed Krebs solution at 32°C. Gastric-projecting DMV neurons were identified based on fluorescence, and electrophysiological recordings were made from identified neurons under bright-field illumination using patch pipettes of 2 to 4 MΩ filled with a potassium gluconate intracellular solution (in mM: 128 potassium gluconate, 10 KCl, 0.3 CaCl_2_, 1 MgCl_2_, 10 HEPES, 1 EGTA, 1 NaATP, and 0.25 NaGTP adjusted to pH 7.35) and a single-electrode voltage-clamp amplifier (Axopatch 200B; Molecular Devices). Data were filtered at 2 kHz, digitized via a Digidata 1440 interface, and stored and analyzed on a PC with pClamp 10 software (Molecular Devices). Recordings with a series resistance of more than 20 MΩ were excluded from the study.

For recordings of eEPSCs, a bipolar stimulating electrode with a tip separation of approximately 125 μm (World Precision Instruments [WPI]) placed on the adjacent NTS eEPSCs using electrical stimuli (10–500 μA, 0.05–1.0 ms) was applied every 10 seconds throughout the recording to evoke submaximal currents in neurons voltage-clamped at –50 mV and +20 mV. In the continued presence of the GABAergic antagonist, bicuculline (30 μM), the amplitude of eEPSCs in the absence and presence of memantine, DHK, AP5, and the AMPA receptor antagonist, DNQX, were used to examine the effects of extrasynaptic NMDAR activation on NMDA-mediated currents. All drugs were prepared fresh and diluted in Krebs solution immediately before use and used at concentrations determined previously to be effective.

Glutamatergic mEPSCs (i.e., action potential–independent neurotransmitter release) were examined by voltage clamping neurons at –50 mV in the presence of tetrodotoxin (TTX; 0.3 μM) and bicuculline (30 μM). The amplitude, frequency, and charge transfer (area × frequency) of mEPSCs in the absence or presence of the synaptic NMDAR–selective antagonists, AP5 (25 μM) and MK801 (5 μM); the extrasynaptic NMDAR antagonist, memantine (30 μM); the NR2B subunit–containing (predominantly extrasynaptic) NMDA receptor antagonists, ifenprodil (3 μM) and ConG (0.6 μM); and the glutamate uptake inhibitor, DHK (30 μM) were examined using MiniAnalysis (Synaptosoft).

For the characterization of action potential firing rate, neurons were current-clamped at a holding potential, which allowed an action potential firing rate of approximately 1 Hz, prior to AP5, memantine, ifenprodil, ConG, or DHK application. Antagonists were applied for a period of time sufficient for the response to reach plateau or at least 5 minutes if a response was not observed. For the characterization of cell-attached firing rate, neurons were allowed a 5-minute recovery period after forming a GΩ seal prior to recording basal firing rate over a 2-minute period.

### In vivo recordings of gastric tone and motility.

Male rats were anesthetized with Inactin (Thiobutabarbital; 125–150mg/kg, i.p.) to a deep plane of anesthesia (abolition of foot pinch withdrawal reflex). A tracheal catheter was fitted and an abdominal laparotomy performed to expose the anterior stomach. Miniaturized strain gauges (AT Engineering) were attached to the ventral surface of the corpus and antrum in alignment with circular smooth muscle, as described previously (76). The strain gauge leads were exteriorized before the abdominal incision was closed. Rats were then placed in a stereotaxic frame (Kopf Instruments) and body temperature was maintained at 37°C via heating pad. The strain gauge signal was filtered (AT Engineering; low pass cutoff 0.5 Hz) and amplified (QuantaMetrics EXP CLSG-2) and recorded on a computer using Axotape 10 software (Molecular Devices).

After placement on the stereotaxic frame, the brainstem was exposed via blunt dissection, the meningeal membranes above the vagal trigone were removed, and the exposed brainstem covered with warmed saline during a recovery period of at least 90 minutes. A borosilicate micropipette (~20 μm tip diameter) was lowered into the left DVC at the coordinates (in mm) +0.4 to 0.5 rostro-caudal from calamus scriptorius, +0.2 to 0.4 medio-lateral from midline, and –0.6–0.65 dorso-ventral from the brainstem surface. Drugs were prepared fresh daily and dissolved in PBS (in mM: 115 NaCl, 75 Na_2_HPO_4_, and 7.5 KH_2_PO_4_) and microinjected in 60 nL volumes using a Picospritzer (Toohey Co.) over a period of approximately 60 seconds, or applied to the fourth ventricle in 2 μL volumes. The effects of drug application on corpus and antrum motility and tone were measured as described previously (76).

After experimentation, rats were euthanized and perfused transcardially with saline followed by 4% paraformaldehyde in PBS. The brainstems were removed and postfixed for 3 days in 4% paraformaldehyde plus 20% sucrose. The brainstems were then frozen and cut in 50 μm sections throughout the rostro-caudal extent of the DVC using a freezing microtome for post hoc verification of microinjection sites and using a Nikon E400 microscope.

### Fourth ventricular cannula placement.

Rats (*n =* 18; *n =* 8 males, 10 females) were anesthetized with a cocktail of ketamine, xylazine, and acepromazine (80 mg/kg, 1.6 mg/kg, and 5 mg/kg, respectively, i.m.) until a deep plane of anesthesia was attained (abolition of the foot pinch withdrawal reflex). Rats were placed on a stereotaxic frame and the skull was exposed via blunt dissection. Guide cannulas (7.9 mm length, 22 gauge, Plastics One) were placed above the fourth ventricle, 2.5 mm anterior to the occipital suture, on the midline, 6.0 mm below the skull surface. The cannulae were affixed to the skull with 3 screws (0–80 × 3–32, Plastics One) and dental cement (Stoelting Co.) and closed with obturators (0.014–0.36 mm, Plastics One) after suturing. Rats were returned to their home cage (singly housed) and administered daily fourth ventricular (i.c.v.) PBS (0.1 M; in mM: 115 NaCl, 75 Na_2_HPO_4_, 7.5 KH_2_PO_4_) to maintain cannula patency and to allow for acclimation to handling. Rats were allowed a minimum of 4 days of recovery prior to experimentation. After 5 days of HFD exposure, all rats were euthanized and the cannula placement was confirmed with i.c.v. administration of the dye, cresyl violet, and gross visual inspection.

### siRNA and scrambled RNA microinjection.

Rats (*n =* 16; 9 males, 7 females) were anesthetized, placed on a stereotaxic frame, and the skull exposed as described above. The brainstem was exposed via blunt dissection, the meningeal membranes above the vagal trigone were removed, and brainstem exposed. A borosilicate micropipette (~20 μm tip diameter) was used to microinject siRNA (GRIN2B; Ambion In Vivo siRNA s127810; Thermo Fisher Scientific) or scrambled RNA (Ambion In Vivo Negative Control). For both siRNA and scrambled RNA, 10 μg RNA plus 1 μg DOTAP liposomal transfection reagent (Millipore Sigma) were added per 1 μL distilled water (*n =* 7 and 9, respectively) into the left and right DVC at the coordinates (in mm) +0.4 to 0.5 rostro-caudal from calamus scriptorius, +0.2 and 0.4 medio-lateral from midline, and –0.6 to 0.65 dorso-ventral from the brainstem surface. Injectate was prepared daily and microinjected in 100 nL volumes using a Picospritzer (Toohey Co.) over a period of approximately 2 minutes. Two injections (medial and lateral) were made in the left and right DVC with 10 minutes of rest between injections. The overlying musculature and skin were sutured and rats returned to their home cages (singly housed). Rats were weighed daily and were used for experiments after 3–5 days once they had reestablished their presurgical weight.

### Measurement of GRIN2B subunit mRNA.

Rats (*n =* 5 scrambled RNA, 3 males, 2 females; *n =* 4 siRNA, 2 males, 2 females) were anesthetized with isoflurane (5% in air) before euthanasia via administration of a bilateral pneumothorax. The brainstem was removed and sliced (400 μm thickness) as described above for electrophysiological recordings. Slices were placed on glass slides with bilateral micropunches taken from DMV and the adjacent hypoglossus as control tissue. Samples were stored at –80°C. Micropunches were homogenized with QIAGEN TissueLyser II. Total RNA was extracted using the RNeasy Lipid Tissue Mini Kit (QIAGEN) with DNase treatment. RNA was quantified via spectrophotometry with Nanodrop 1000, and approximately 80 ng of RNA was quantitatively converted into cDNA (High Capacity cDNA Reverse Transcription Kit with RNase Inhibitor; Applied Biosystems) using a Veriti 96-well thermal cycler (Applied Biosystems). cDNA was prepared for qPCR using TaqMan Universal Master Mix II and TaqMan Gene Expression Assays (GRIN2B: Rn00680474-_m1, Thermo Fisher Scientific) and cycled into a QuantStudio 12k flex thermal cycler (Applied Biosystems) according to the manufacturer’s instructions. All samples were prepared in triplicate with data normalized to β-actin levels (β-actin: Rn00667869_m1, Thermo Fisher Scientific) and expressed as fold change using the 2^–ΔΔCT^ method.

### Gastric emptying measurement.

The gastric emptying rate for a solid meal was measured in male rats (*n =* 6) using the ^13^C octanoic acid breath test. After 3 days of acclimation to the testing chamber (1 rat per chamber) and pancake meal, rats were fasted overnight with ad libitum access to water before placement in testing chamber. Testing chambers had a controlled air flow rate with CO_2_ levels maintained between 1000 and 2000 ppm. After 60 minutes of baseline recordings, rats were given 1 g of pancake (Pillsbury Homestyle Pancakes, General Mills) treated with 4 μL of [^13^C]-octanoic acid (Cambridge Isotope Laboratories, Inc.). Rats that did not consume the entire pancake within 5 minutes were removed from the study. Air from the 4 testing chambers was automatically sampled one at a time for 30 seconds each at a sample rate of 1 Hz for 8 hours. The air sample was analyzed by off-axis integrated cavity output spectroscopy using a multiple input unit and carbon dioxide carbon isotope analyzer (Los Gatos Research).

For each 30-second time group of data, the first 10 seconds were removed to ensure a complete flush of the previous air sample from the tubing (76). The remaining 20 seconds of data were averaged for a single data point at a given time. The concentration of ^13^CO_2_ was then calculated and expressed as change over the baseline. The change of concentration of ^13^CO_2_ versus time (t) was fitted by a nonlinear regression curve with the following equation: y = at^b^e–ct where y is the percentage of the ^13^C excretion in the breath per hour (t) and a, b, and c are regression constants estimated for each breath versus time curve. The gastric half-emptying time (T_1/2_) was calculated from a numerical integration procedure using an inverse γ function.

Three baseline measurements were taken with a minimum of 4 days between testing. Rats were exposed to an HFD and gastric emptying was measured at 1 and 4 days of HFD exposure.

### Gastric compliance measurement.

Male rats (*n =* 6 control, *n =* 8 HFD) were anesthetized with Inactin (Thiobutabarbital; 125–150mg/kg, i.p.) to a deep plane of anesthesia (abolition of foot pinch withdrawal reflex). An abdominal laparotomy was performed and a latex balloon (maximal volume 2 mL) was inserted into the stomach via an incision made in the duodenum. After placement, the balloon was maximally inflated (2 mL; 1 minute) before deflation. After 10 minutes of recovery, the balloon was then inflated in 0.2 mL increments for 1 minute each, and the balloon pressure measured continuously. The signal was filtered (AT Engineering; low pass cutoff 0.5 Hz), amplified (QuantaMetrics EXP CLSG-2), and recorded on a computer using Axotape 10 software (Molecular Devices). The relative pressure was measured at the end of each 1-minute interval throughout the range of volumes measured. Gastric compliance was measured at baseline and 4 days after HFD exposure.

### Food intake measurements.

After placement of fourth ventricular cannulae and surgical recovery, food intake and body weight were measured twice daily within 1 hour of lights on-off. PBS (2 μL) was i.c.v. administered once daily within 1 hour before the lights-off cycle. After 4 days of baseline measurement and acclimation to daily i.c.v. injection, rats (*n =* 13) were exposed to an HFD. On the day of HFD exposure, 1 group (*n =* 6) began daily i.c.v. memantine administration (50 pmol/2 μL in PBS), while the remainder (*n =* 7) continued to receive PBS. An additional group (*n =* 5) remained on a control diet throughout daily i.c.v. memantine treatment (50 pmol/2 μL in PBS).

### Statistics.

For electrophysiological results, each neuron served as its own control; the response of any neuron was assessed before and after drug application using a paired, 2-tailed Student’s *t* test. The magnitude of response to an individual drug was compared across groups with an unpaired Student’s *t* test, 1-way ANOVA with post hoc Bonferroni test, or Dunnett’s test for multiple comparisons. Intergroup comparisons were made using the χ^2^ test. Neurons were divided into responsive and nonresponsive neurons based on their pharmacological phenotype. A responsive neuron was considered one in which a drug induced a change in action potential firing rate greater than 25%, a change in eEPSC amplitude of more than 20%, and a change in mEPSC frequency of more than 20%; all results (responsive and nonresponsive) are reported as the mean ± SEM, with significance defined as *P* less than 0.05.

For in vivo recordings, strain gauges were calibrated prior to use and drug-induced effects on gastric motility and tone were extrapolated from the average calibration value. Although basal gastric tone was not adjusted to a fixed value, the gastric circular muscle provided a basal tension of approximately 500 mg. Corpus and antrum tone are reported as absolute values relative to baseline because of individual variation in animal size and surgical placement of the strain gauges, which may lead to minor variations in absolute values. Each rat served as its own control, and corpus and antrum motility were calculated using the following formula: Motility index (%) = 100 × [(N_1_ × 1) + (N_2_ × 2) + (N_3_ × 4) + (N_4_ × 8)]/t where N_x_ = the number of motility peaks in a given force range and t = time period over which motility was measured. With the assumption that the absence of motility produced a 0 mV signal, the peak-to-peak motility waves reflected N_1_ = 25–50 mg, N_2_ = 51–100 mg, N_3_ = 101–200 mg, and N_4_ = >201 mg.

Each rat served as its own control, and the gastric responses were assessed before and after DVC microinjection using a paired Student’s *t* test. Intergroup comparisons were made using the unpaired Student’s *t* test or 1-way ANOVA with post hoc Bonferroni analysis. Thyrotropin-releasing hormone (TRH) was used to confirm the microinjection site, and only rats that demonstrated an increase in gastric tone and motility in response to TRH microinjection were included in the statistical analyses. A physiologically significant response was considered a change in motility of at least 25%, with significance defined as *P* less than 0.05.

Caloric intake is expressed as percentage of baseline, with baseline defined as the average caloric intake measured over 3 days of control diet intake. The AUC for normalized caloric intake was calculated using GraphPad Prism and compared using 1-way ANOVA with post hoc Dunnett’s test. Results are expressed as mean ± SEM of the indicated sample size (*N*; cells or rats), with significance defined as *P* less than 0.05.

mRNA data are expressed as fold change using the 2^–ΔΔCT^ method and compared between scrambled RNA– and siRNA-injected rats separately within each nucleus (DMV versus hypoglossus) using Student’s unpaired *t* test, with results expressed as mean ± SEM and significance defined as *P* less than 0.05. Data were analyzed using QuantStudio 12k Flex software (Applied Biosystems) and GraphPad Prism 6.

### Study approval.

All experiments were conducted with the approval of the Penn State University College of Medicine IACUC and in accordance with NIH regulations. Reporting of animal experiments conforms to the principles and regulations for animal experiment reporting and ethics and conforms with the ARRIVE guidelines (77).

## Author contributions

CC, RAT, and KNB conceived and designed research; CC, RAT, ACA, and KNB performed experiments; CC, RAT, ACA, and KNB analyzed data; CC, AT, ACA, and KNB interpreted results of experiments; CC and KNB prepared figures; CC and KNB drafted the manuscript; CC, RAT, ACA, and KNB edited and revised the manuscript; CC, RAT, ACA, and KNB approved the final version of the manuscript.

## Supplementary Material

Supplemental data

## Figures and Tables

**Table 1:**
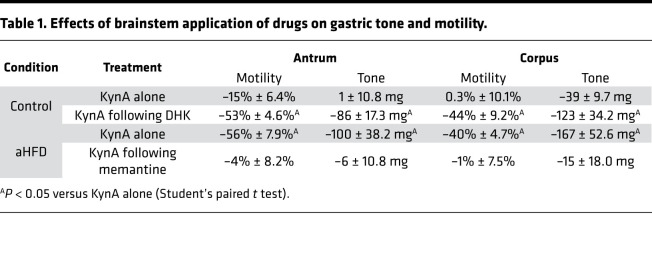
Effects of brainstem application of drugs on gastric tone and motility.

## Version 1 03/25/2021

In-Press Preview

## Version 2 05/10/2021

Electronic publication
