# Supplementary material for: DMV extrasynaptic NMDA receptors regulate caloric intake in rats
Source: JCI Insight. 2021 May 10;6(9):e139785. doi: 10.1172/jci.insight.139785 (PMC8262316; doi:10.1172/jci.insight.139785)
Supplement: Supplemental data [file jciinsight-6-139785-s181.pdf]

Supplementary Table 1: Effects of glutamatergic antagonists on eEPSC properties in control and aHFD DMV neurons

Condition	Treatment	eEPSC Measurement		
		**Amplitude (pA)**	**Decay time (ms)**	**Area (pA.ms)**
Control	Baseline	189±58.3	5.1±0.32	1214±156.9
	Memantine	210±70.7	4.7±0.19	1285±184.2
	Baseline	174±51.5	8.2±2.05	1446±335.3
	Ifenprodil	173±51.4	7.4±1.78	1394±232.3
aHFD	Baseline	142±31.9	4.5±1.50	1685±329.6
	Memantine	151±39.3	4.7±1.78	1628±366.9
	Baseline	178±33.0	5.1±1.75	3523±1448.6
	Ifenprodil	194±42.5	5.3±2.08	2871±1122.5

No significance via Students paired t-test

Supplementary Table 2: Effects of glutamatergic antagonists on mEPSC properties in control and aHFD DMV neurons

Condition	Treatment	mEPSC Measurement		
		Frequency (events.s^{-1})	Amplitude (pA)	Charge Transfer (pA.ms)
Control	Baseline	5.6±2.02	24.6±1.92	196.1±106.1
	AP5 alone	5.3±2.02	23.0±1.84	169.9±90.36
	Baseline	2.4±0.90	25.9±2.19	157.9±54.86
	Ifenprodil alone	2.3±0.92	26.0±1.81	167.0±62.94
	Baseline	2.1±0.89	21.1±1.98	168.3±75.57
	Memantine	1.2±0.29	19.7±1.60	89.3±19.78
	Memantine + AP5	1.4±0.39	19.2±1.58	90.7±24.6
	Baseline	2.9±0.78	19.6±2.85	256.5±76.18
	DHK	3.2±0.88	20.1±1.16	276.7±90.03
	DHK + AP5	**2.2±0.65****	18.6±1.11	**188.5±62.54****
	Scrambled RNA baseline	3.0±0.84	27.5±2.39	239±81.3
	AP5 alone	1.9±0.61	28.3±2.84	146±45.0
aHFD	Baseline	3.4±0.73	22.6±1.41	201.1±47.03
	AP5 alone	**2.2±0.50***	24.6±1.61	**133.1±31.25***
	Baseline	2.5±0.87	27.2±4.84	279.2±92.37
	Ifenprodil	**1.8±0.61***	28.2±3.86	**218.8±84.42***
	Ifenprodil + AP5	1.6±0.75	26.6±0.75	197.4±79.01
	Baseline	4.0±1.35	27.7±2.01	314.1±110.6
	Memantine	3.3±1.16	24.7±2.48	257.7±93.0
	Memantine + AP5	3.0±1.06	25.4±2.55	246.4±94.68
	Baseline	0.87±0.80	22.5±1.12	86±22.8
	DHK	0.85±0.32	21.5±1.22	86±33.1
	DHK + AP5	**0.40±0.09****	18.3±1.14	**30±7.3****
	Baseline	2.4±0.40	27.3±0.81	211.3±50.96
	ConG	**1.4±0.25***	27.0±0.71	**136.2±31.77***
	ConG + AP5	1.3±0.19	26.2±0.49	124.9±27.98
	Baseline	3.5±0.68	26.7±4.9	283±58.1
	MK801	**2.1±0.58***	25.4±4.6	**182±49.8***
	MK801 + memantine	2.2±0.60	26.7±4.0	191±64.3
	siRNA baseline	4.6±1.21	34.4±3.0	480±128.3
	AP5 alone	4.5±1.18	35.4±8.7	446±114.8
	siRNA baseline	6.0±1.26	37.7±3.65	544±123.9
	DHK	6.1±1.28	37.7±3.49	563±142.1
	DHK + AP5	5.9±1.21	37.2±3.10	559±136.4

*P<0.05 vs baseline; **P<0.05 vs DHK (Students paired t-test)

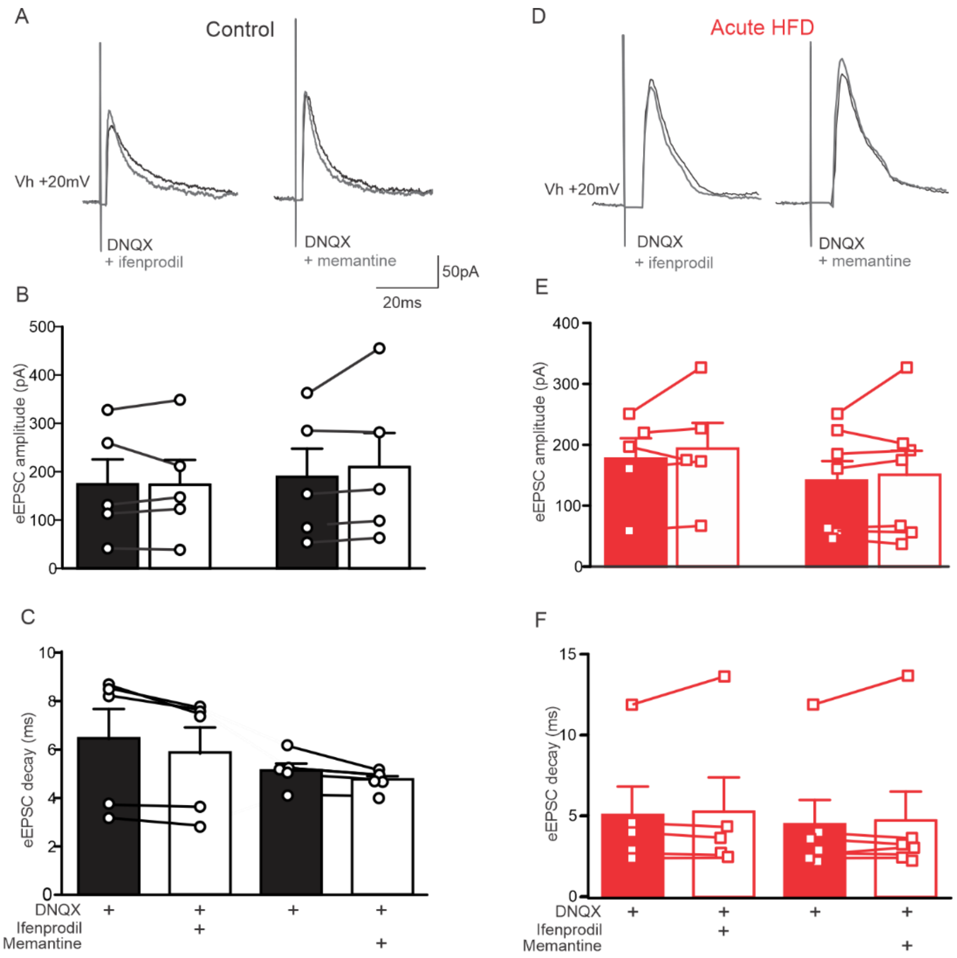

Supplemental Figure 1: Memantine and ifenprodil are selective for extrasynaptic NMDAR and do not affect synaptic NMDA-mediated current.

A: eEPSCs from control DMV neurons voltage-clamped at +20mV in the presence of DNQX. Note that application of ifenprodil (left) or memantine (right) had no significant effect on eEPSC amplitude.
B. Graphical summary of the amplitude of eEPSCs in control DMV neurons in the presence of DNQX. Perfusion of ifenprodil (left; N=5 cells, 3 rats) and memantine (right; N=5 cells, 3 rats) had no significant effect on eEPSC amplitude.
C. Graphical summary of the decay kinetics of eEPSCs in control DMV neurons in the presence of DNQX. Perfusion of ifenprodil (left; N=5 cells, 3 rats) and memantine (right; N=5 cells, 3 rats) had no significant effect on eEPSC current decay.
D eEPSCs from aHFD DMV neurons voltage-clamped at +20mV in the presence of DNQX. Note that application of ifenprodil (left) and memantine (right) had no significant effect on eEPSC amplitude.
E. Graphical summary of the amplitude of eEPSCs in aHFD DMV neurons in the presence of DNQX. Perfusion of ifenprodil (left; N=5 cells, 3 rats) and memantine (right; N=7 cells, 3 rats) had no significant effect on eEPSC amplitude.
F. Graphical summary of the decay kinetics of eEPSCs in aHFD DMV neurons in the presence of DNQX. Perfusion of ifenprodil (left; N=5 cells, 3 rats) and memantine (right; N=7 cells, 3 rats) had no significant effect on eEPSC current decay.
No significance via Students paired t-test

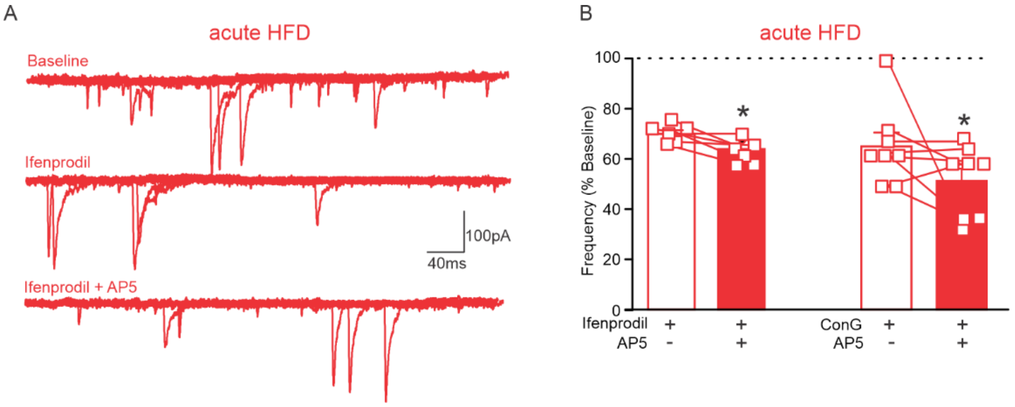

Supplemental Figure 2: Inhibition of NR2B subunit-containing (extrasynaptic) NMDA receptors attenuates the $NMDAR_s$-mediated decrease in glutamatergic currents observed following aHFD exposure

A. Six overlapping consecutive traces from gastric-projecting DMV neurons following aHFD exposure voltage-clamped at -50mV illustrating mEPSCs. Ifenprodil (3μM; middle; N=6/6 cells. 4 rats) significantly decreases mEPSC frequency and subsequent perfusion with the synaptic NMDA receptor antagonist, AP5 (25μM; bottom) had no significant effect on mEPSC frequency.
B. Graphical summary of the effects of ifenprodil (left) and ConG (right) with AP5 on mEPSC frequency in aHFD gastric-projecting DMV. Note that AP5 does not significantly affect mEPSC frequency following application of ifenprodil (N=6 cells, 4 rats) or ConG (N=8 cells, 3 rats). Baseline represented by dashed line (100%). *$P<0.05$ vs. baseline (Students paired t-test).

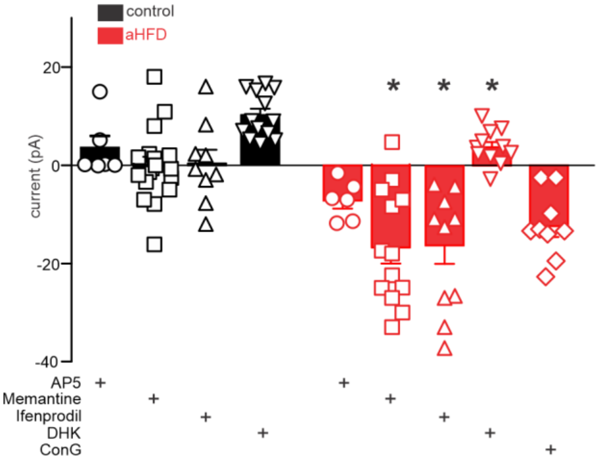

Supplemental Figure 3: Inhibition of $NMDAR_{ex}$ induces an outward current in aHFD DMV neurons

Graphical summary of the changes in holding current in control (black) and aHFD (red) DMV neurons following perfusion with NMDAR-selective antagonists. AP5 (25μM) had no effect on holding current in control (black; N=6 neurons, 3 rats) or aHFD (red; N=6 neurons, 2 rats) neurons, whereas memantine (30μM), ifenprodil (3μM) and ConG (0.6μM) induced an outward current in aHFD (N=13 neurons from 5 rats; N=11 neurons from 6 rats; N=8 neurons from 3 rats, respectively) but neither memantine nor ifenprodil had any effect in control neurons (N=19 neurons from 8 rats; N=9 neurons from 4 rats). In contrast, DHK (30μM) induced an inward current in control (N=13 neurons, 4 rats), but not aHFD (N=11 neurons, 3 rats) DMV neurons (*P<0.05 vs control).
No significance via Students paired t-test vs. baseline.

.

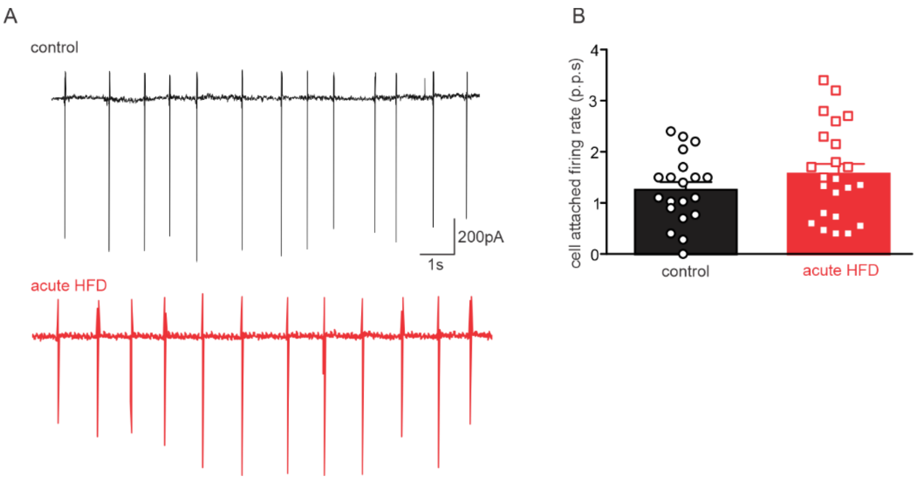

Supplemental Figure 4: Cell attached firing rats in control and aHFD DMV neurons

A. Representative traces of cell attached firing rats in control (black) and aHFD (red) DMV neurons.
B. Graphical summary of the cell attached firing rats in control (black; N=20 neurons, 6 rats) and aHFD (red; N=23 neurons,10 rats) DMV neurons. Note that aHFD exposure does not alter spontaneous firing rate in DMV neurons.
No significance via Students paired t-test
